# Transmitted HIV drug resistance and subtype patterns among blood donors in Poland

**DOI:** 10.1038/s41598-021-92210-2

**Published:** 2021-06-17

**Authors:** Miłosz Parczewski, Ewa Sulkowska, Anna Urbańska, Kaja Scheibe, Karol Serwin, Piotr Grabarczyk

**Affiliations:** 1 Department of Infectious, Tropical Diseases and Immune Deficiency, Pomeranian Medical University, Arkońska 4, 71-455 Szczecin, Poland; 2 Institute of Haematology and Transfusion Medicine in Warsaw, Warsaw, Poland

**Keywords:** Clinical microbiology, Infectious-disease diagnostics, Virology

## Abstract

Surveillance on the HIV molecular variability, risk of drug resistance transmission and evolution of novel viral variants among blood donors remains an understudied aspect of hemovigilance. This nationwide study analyses patterns of HIV diversity and transmitted resistance mutations. Study included 185 samples from the first time and repeat blood donors with HIV infection identified by molecular assay. HIV protease, reverse transcriptase and integrase were sequenced using population methods. Drug resistance mutation (DRM) patterns were analyzed based on the Stanford Interpretation Algorithm and standardized lists of transmitted mutations. Phylogeny was used to investigate subtyping, clustering and recombination patterns. HIV-1 subtype B (89.2%) followed by subtype A6 (7.6%) were predominant, while in three (1.6%) cases, novel recombinant B/A6 variants were identified. Non-B variants were more common among repeat donors (14.5%) compared to the first time ones (1.8%), *p* = 0.011, with higher frequency (9.9%) of A6 variant in the repeat donor group,* p* = 0.04. Major NRTI DRMs were observed in 3.8%, NNRTI and PI in 0.6% and INSTI 1.1% of cases. Additionally, E157Q polymorphism was observed in 9.8% and L74I in 11.5% of integrase sequences. Transmission of drug resistance among blood donors remains infrequent. Subtype patters increase in complexity with emergence of novel intersubtype A6B recombinants.

## Introduction

Molecular surveillance allows to model the evolution of the HIV epidemics, while investigation of the drug resistance, subtype variability and identification of the new recombinant variants remains a valuable tool to inform public health strategies^1,2^. Monitoring of the drug resistance patterns, especially in the evolving landscape of the antiretroviral drugs, rapid antiretroviral treatment introduction and expanding use of the pre-exposure prophylaxis remains key to maintain virologic efficacy of combined antiretroviral treatment (cART) and preserve future treatment options^3–6^. Studies on the transmission of the HIV drug resistance have provided data to prioritize the selection of the first and second-line antiretroviral drugs^7^. Importantly, molecular characteristics of the HIV strains observed among blood donors may reflect circulation patterns in the untested general population, generally unaware of the transmission risk, which in turn is of the primary importance to the blood safety^8^. Additionally, amplification efficiency and sensitivity of the molecular assays used for the blood screening may differ for novel variants and resistance mutations^9–11^.

In Poland, HIV epidemics has evolved to the primarily sexually transmitted, with the highest transmission risk among men-who-have-sex with men and increasing number of new infections despite generally low level prevalence^12^. Since the beginning of epidemics until the end of 2019 HIV was diagnosed in 25 544 cases with 1429 registered AIDS deaths. Of note, in the recent years increasing number of immigrant population from the regions with the expanding HIV epidemics (Ukraine, Russia and Belarus) have been registered in the country however, records on the nationality among newly diagnosed individuals are commonly missing. So far, the most prevalent HIV-1 strain was subtype B (86.9%) followed subtype A (5.2%), D (3.5%) and C (1.8%) and infrequent identification of the recombinant forms (~ 2.1%)^13^. Among treatment naïve cases transmitted drug resistance mutations (DRM) were observed in ~ 9% of cases in the national wide studies (5.8% for nucleoside reverse transcriptase, 1.2% for non-nucleoside reverse transcriptase, and 2.0% for protease mutations) and up to ~ 12% in local cohorts with no transmitted major integrase mutations observed so far^14–16^. Additionally, frequency of transmission clusters ranges from 20–30% which indicates high likelihood of HIV epidemics expansion in the future^17,18^.

Among blood donors the frequency of detected HIV infection in Europe remains low, however constant hemovigilance is required to identify the breakthrough transmissions in the pre-seroconversion period^9–11^. In general, currently implemented blood testing strategy based on nucleic acid amplification testing has proven to be effective^9^. The average frequency of HIV detection per 100.000 (confidential limit, 95% Cl) among Polish blood donors in period 2005–2018 was 6.26 (5.77–6.85) for seropositive and 0.28 (0.19–0.42) for seronegative infections. In total the frequency of infected donors and donations per 100.000 was 6.56 (6.03–7.14) and 3.3 (3.04–3.59), respectively. In this period a slight increase in infection rate was noticeable and the HIV infection rate was significantly higher among the first time than in repeat blood donors. All seronegative HIV-NAT positive donors were men and all but one were repeat donors, however until now *Look-back* procedure has not documented any HIV transmission via blood component transfusion. Mathematical models estimate the risk of infectious transfusions at 0.16 to 0.49 per million, depending on screening format (sensitivity) and type of blood component (plasma volume)^19^.

In this considerably sized nationwide study, HIV diversity, patterns of transmitted drug resistance and clustering among Polish blood donors were analysed. This is the first study informing on the molecular variability of HIV in this group, identifying risk of DRM transmission and evolution of novel viral variants, which is of the utmost importance for the blood services.

## Methods

### Study group

For this study 235 samples with HIV infection confirmed by the molecular test, diagnosed during blood donation, were collected, of these 185 (78.7%) samples were successfully sequenced and included in the analyzed dataset. Sampling period spanned from 2009 to 2017 and included samples collected from the entire Poland, with positive samples obtained from the following blood collection centers: Białystok, Bydgoszcz, Gdańsk, Kalisz, Katowice, Kielce, Kraków, Lublin, Łódź, Olsztyn, Opole, Poznań, Racibórz, Radom, Rzeszów, Słupsk, Szczecin, Wałbrzych, Warszawa, Wrocław, Zielona Góra and military blood transfusion service. In this period in total 387 blood donors with HIV positive nucleic acid amplification test (NAAT) were identified (370 donors with detectable both HIV-RNA and anti-HIV antibody and 17 in the pre-seroconversion period with negative HIV serology, supplemental Table 1); therefore the study represents 60.7% of the total number of HIV NAAT positive blood donors. For HIV serological markers screening in blood donors 3rd or 4th generation CE-marked assays were used. All blood donations were tested in individual donations (IDT) with TMA based assays: initially with Procleix Ultrio Plus and later with Procleix Ultrio Ellite (Gen-probe, USA) or with real-time PCR in minipools of 6 donations (MP6) using MPX system (Roche, USA). Donations reactive in screening were shipped to the reference laboratory at the Institute of Haematology and Transfusion Medicine in Warsaw for confirmatory tests that included Western/immuno blot and RNA HIV testing. Western/immuno blot analyses were performed using commercial assays that were changed periodically: HIV BLOT, Genelabs® Diagnostics (Singapore); INNO-LIA™ HIV I/II Score, Innogenetics (Belgium), HIV BLOT MP Diagnostics (Singapore). NAAT test used were changed over the years to reflect technological progress and increasing sensitivity: Firstrly, Cobas Ampliscreen HIV-1 v 1.5 Roche and Procleix Ultrio Plus were used, then Gen-probe USA/ and later Procleix Ultrio Elite, Gen-probe USA/ Confirmatory PCR Kit HIV-1 v 1.2 GFE Blut Germany. Newer assays were able to detect 2–3 HIV genome regions; each sample was confirmed using at least two methodologies, as noted above.

The study was approved by the bioethical committee of Pomeranian Medical University, Szczecin, Poland (approval number BN-001/34/04). The research was conducted in accordance with the Declaration of Helsinki. All data were anonymized. At the time of consent for the blood collection procedure the participant provides an informed consent related to the molecular analyses of the presence of the viral pathogens and subsequent analyses including molecular epidemiology of the viruses. Such an informed consent was obtained from all subjects included in the study. HIV-1 viral loads were not available, as the screening in blood collection centers is performed using qualitative HIV assay.

Data collected included type of donation (first time vs. repeated), gender, age, nationality, time since last donation (if repeated donor) and HIV infection (Fiebig) stage^20,21^. Fiebig stage was assessed based on the HIV molecular markers (HIV-RNA), p24 antigen, enzyme immunoassay reactivity and Western-blot patterns.

Before donation, prospective donors were screened for high-risk activities through a predonation, paper-based questionnaire. A brief physical examination was also performed. This procedure allows to determine whether the candidate is suitable for donating blood. Based on this evaluation, prospective donors could be temporarily or permanently deferred from donating blood. Donors also were deferred based reported certain risky behaviors including high-risk sexual activity (sex with multiple partners or with unknown partner (-s), having a sexual partner who injects drugs, commercial sex work, reported contact with person infected with HIV, HB, HCV or and *Treponema pallidum*), a history of criminal arrests or detention, intravenous drug use, exposure to blood from another person, selected medical (surgery, transplantations, gastroscopy etc.) or cosmetical (piercing, tattoo etc.) procedures. There were no HCV or HBV coinfected individuals in the analyzed group.

### Sequencing

HIV-1 protease (PR) and reverse transcriptase (RT) genotyping and sequence assembly was performed using Viroseq 2.9 genotyping kit (Abbott Molecular, Abbott Park, IL) according to manufacturer’s protocol providing a sequence of 1302 base pair (b.p.) long with inclusion of 1–99 codons in the PR and 1–335 in the RT. Additionally, HIV-1 integrase (IN) region (866 b.p., codons 1–288) was amplified and sequenced with reagents and conditions specified by Laethem et al.^22^. Amplicons obtained by the nested PCR method were used for sequencing by standard techniques with BigDye technology on an ABI 3500 platform (Applied Biosystems, Foster City, CA). Integrase sequence assembly was performed with the Recall online tool^23^. PR/RT sequencing was successful for 159 (85.9%) cases, integrase for 174 (94.1%) samples. Final dataset included 148 (78.9%) patients with both PR/RT and IN sequence, 11 (5.9%) only with PR/RT and 26 (14.1%) only with IN sequence.

### Subtyping and drug resistance interpretation

Initial subtyping was performed using automated genotyping software (REGA genotyping 3.46 tool^24^, based on the obtained PR, RT and IN sequences. Subtyping, including identification of subgroups for the subtype A, was verified using phylogenetic methods with reference sequences with known subtype from the 2018 version of the HIV sequence compendium (Los Alamos National Laboratory) (https://hfv.lanl.gov/content/sequence/HIV/COMPENDIUM/compendium.html) , supplemented with local sequences from the HIV sequence database (http://www.hiv.lanl.gov/components/sequence/HIV/search/search.html). Phylogenetic trees of A-clade were made with a representative number of 178 sequences, comprising 101 references from the LANL-HIV database, 58 unique regional sequences with homology over 95% (based onBLAST analysis), and finally subtype O—as an out-group sequence. Method of maximum likelihood (ML) with approximate likelihood ratio test (aLRT) and Shimodaira–Hasegawa (SH) algorithm was performed among the support of PHYMLv3.0 web server.

For identification of drug resistance mutations Stanford Genotypic Resistance Interpretation Algorithm (https://hivdb.stanford.edu/hivdb/by-sequences/) was used, with classification of drug resistance mutations into major and accessory for PR and IN, as well as nucleoside and non-nucleoside inhibitor drug resistance (NRTI and NNRTI) for RT. Mutations with the scoring $\ge$ 10 for at least one active drug were included in the analyses. Additionally, PR/RT mutations were assessed according to WHO surveillance list^25^, while for integrase strand transfer inhibitor mutations standardized list of INSTI-resistance mutations was used^26^. In the final analyses we have also included the L74M integrase polymorphism as included in the IAS 2019 drug resistance update^27^.

### Phylogenetic analyses

For the phylogenetic relationships, the PR/RT and IN sequences were concatenated (2168 bp length) and aligned with Clustal Omega^28^ software separately for subtype A and B. Subtype C and URFs were excluded from the phylogenetic analyses due to small sample size. Methodology to use concatenated sequences spanning different locations in HIV genome was used previously in numerous studies^29–31^ and HIV subtyping program (https://hivdb.stanford.edu/page/hiv-subtyper). Following the alignment, the optimal tree model was estimated using jModelTest 2.1.10 software for subtype A and subtype B sequences^32^. In both cases, the best fitting model was the GTR with four gamma categories. Rate parameters were as follows, for subtype A and subtype B, respectively: freqA = (0.3975/0.4221), freqC = (0.1581/0.1577), freqG = (0.2240/0.2077), freqT = (0.2205/0.2125), gamma shape parameter 0.4960/0.8810) and proportion of invariant sites of 0.3180/0.4850. Under these parameters three separate Bayesian Monte Carlo Markov Chain analyses were run in triplicates for 50 million of generations in Beast v. 2.0^33^ using Yuole Model with a strict molecular clock using the above coefficients. In this scenario, all parameters achieved the effective sample size (ESS) value above 200. Clustering was assessed using Cluster Picker software with the maximum genetic distances calculated by the program. Clusters were identified for the sequences with the posterior value > 0.9 for external taxonomical units and mean pairwise distances < 0.015^34^. All trees were visualized in Figtree v.1.4.4.

### Statistics

Statistical comparisons were performed using Fisher’s exact and Chi^2^ tests for nominal variables as appropriate. Continuous variables were analysed using the Mann–Whitney *U*-test for nonparametric statistics. Confidence intervals (CI) and interquartile ranges (IQR) were indicated where appropriate. Commercial software (Statistica 11.0PL, Statasoft, Warsaw, Poland) was used for these statistical calculations.

### Sequence data

Sequences from this study have been submitted to GenBank and may be accessed with the following IDs: MZ218761 - MZ218932 and MZ218933 - MZ219089.

## Results

### Overall group characteristics and HIV-1 subtypes

**Figure 1:**
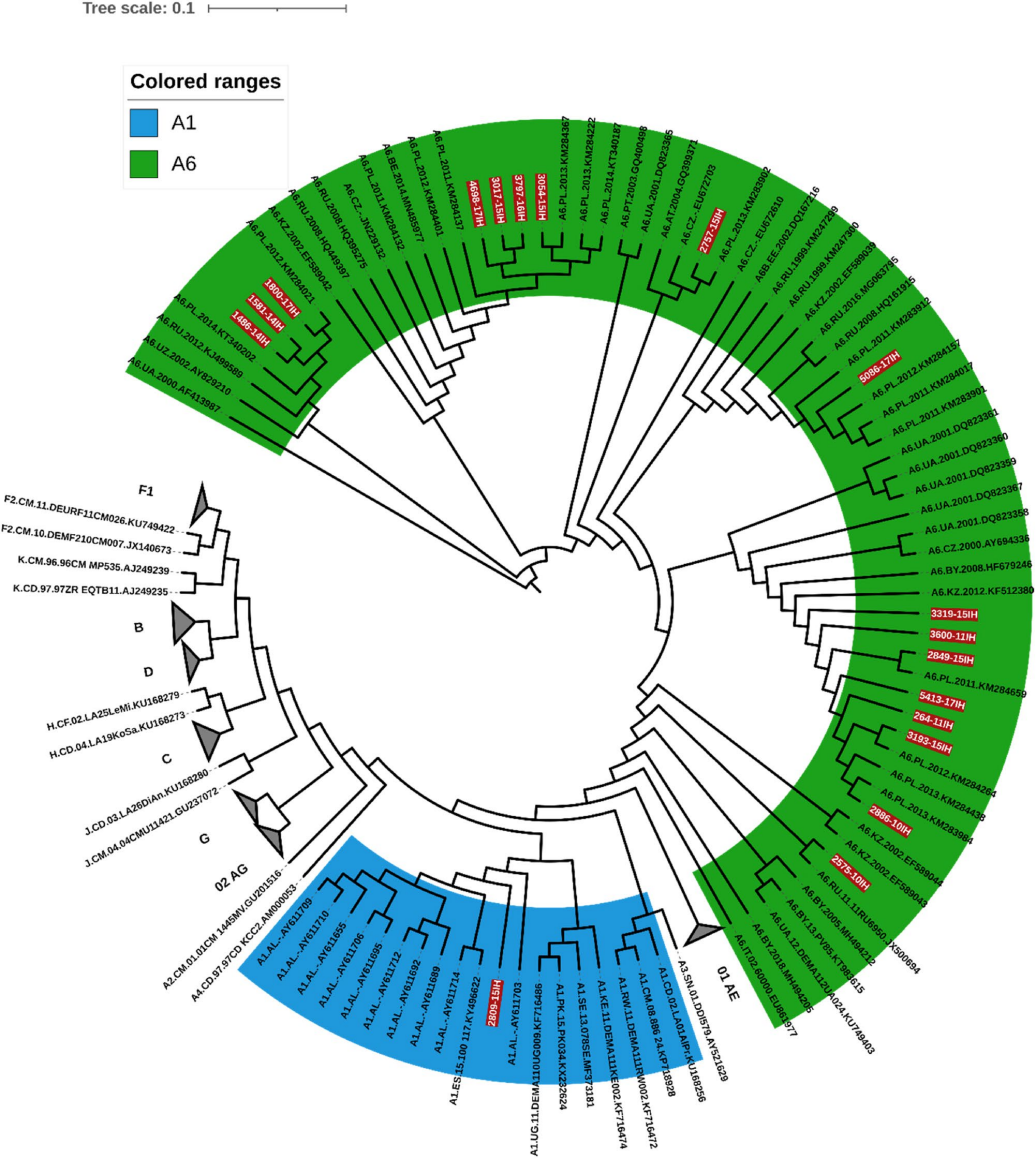
Maximum likelihood tree showing the subtype A identification with the reference sequences from HIV sequence compendium supplemented with A1 and A6 sequences from GenBank. A1 variant is colored in blue, A6 in green.

**Figure 2:**
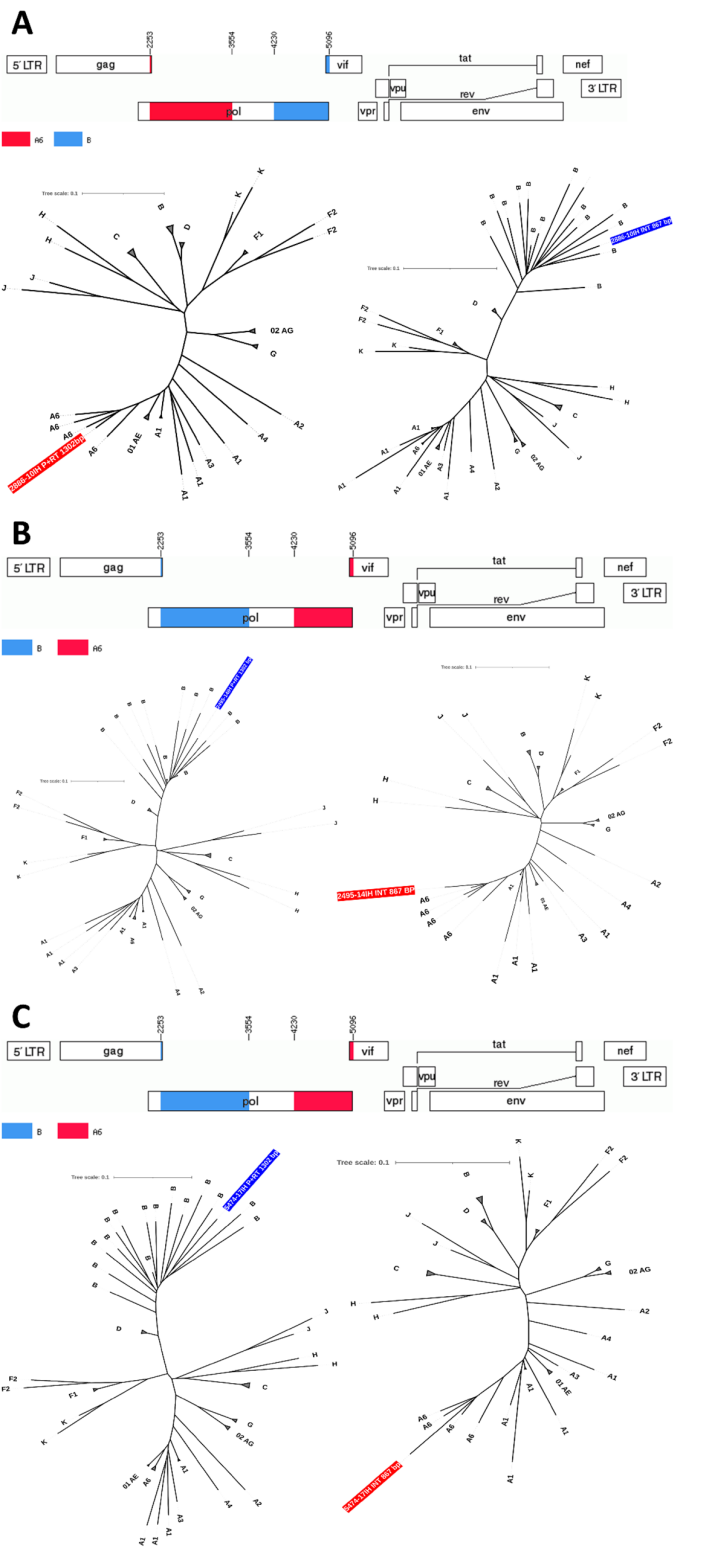
Three unique recombinant form genome maps and the separate phylogenetic ML trees with the corresponding partial protease/reverse transcriptase and integrase sequences confirming the subtype assignment. For the tree reconstruction a dataset obtained from HIV sequence compendium 2018 was used. Multiple branches for the same subtype were collapsed. A. sequence 2886 acquired in the city of Warsaw, B. sequence 2495 acquired in the city of Krakow, C. sequence 5474 acquired in the city of Warsaw.

The studied group included predominantly male individuals (95.7%) with the median age of 29 (IQR: 24–34) years. Of these, 54 (29.2%) individuals were the first time, while 131 (70.8%) were the repeat donors. The most prevalent HIV-1 variant was subtype B (n = 165, 89.2%) followed by subtype A (n = 15, 8.2%) and subtype C (n = 2, 0.1%) (Table 1). Of note, when utilizing the REGA 3.46 on-line automated subtyping tool all subtype A sequences were assigned as A1 variant, while phylogeny with reference sequences confirmed that in fact only one sequence belongs to the A1 subgroup, while the remaining sequences (n = 14, 7.6%) are in fact A6 subtype (Fig. 1). In three cases, novel recombinant variants with breakpoints between the reverse transcriptase and integrase coding region, were found and confirmed phylogenetically (2 sequences with B/A6 and one with A6/B) (Fig. 2).

**Table 1:** Baseline cohort characteristics by first time/recurrent donors.

	First time donor	Recurrent donor	p	Total
*Gender, n (%)*				
Male	50 (92.6)	127 (96.9)	0.18	177 (95.7)
Female	4 (7.4)	4 ( 3.1)		8 (4.3)
Age, median (IQR)	29 (24–34)	28 (25–34)	0.86	29 (24–34)
*Fiebig stage, n (%)*^*#*^				
I	1 (1.9)	7 (5.3)	0.03	8 (4.3)
II	0	2 (1.5)		2 (1.1)
III	0	0		0
IV	0	4 (3.1)		4 (2.2)
V	8 (14.8)	40 (30.5)		48 (25.9)
VI	45 (83.3)	78 (59.5)		123 (66.5)
*HIV subtype, n (%)*				
A1	0	1 (0.8)	0.16	1 (0.5)
A6	1 (1.8)	13 (9.9)		14 (7.6)
B	53 (98)	112 (85.5)		165 (89.2)
C	0	2 (1.5)		2 (1.1)
URF	0	3 (2.3)		3 (1.6)
*Subtype B vs non-B variants*				
Subtype B	53 (98.2)	112 (85.5)	0.011	163 (88.1)
Non-B variants	1 (1.8)	19 (14.5)		20 (11.9)

^#^There were no individuals with Fiebig stage III.

In the group of the recurrent donors median time from the last donation was 226 (IQR: 121–654) days [median 169 (IQR: 68–315) days for the Fiebig I stage, median 93 (IQR:66–121) for II, median 81 (IQR: 42–111) for IV, median: 152 (88–333) for V and median 420 (165–1025) for VI. Median time from the last donation was notably longer for individuals with HIV diagnosed in Fiebig stages I-IV [median time 144 (IQR:66–169] days compared to stages V and VI [median time 241 (IQR: 136–730) days], *p* = 0.003, and Fiebig stages I-V [median time 139 (IQR:79–304)] days vs VI (*p* < 0.0001).

There was a notable difference in the distribution of the Fiebig stages between the first time and repeat donors (*p* = 0.03, Table 1), with Fiebig stage I-IV being more common among repeat donors (n = 13, 9.9%) vs. first time donors (n = 1, 1.9%), *p* = 0.047. Additionally, stages I-V were observed among 40.5% (n = 53) repeat donors vs. 16.7% (n = 9) first time donors (*p* = 0.002).

Interestingly, HIV-1 non-B variants were notably more common among repeat donors (n = 19, 14.5%) compared to the first time ones (n = 1, 1.8%), *p* = 0.011, which was associated with higher frequency of A6 variant in the repeat donor group (n = 13, 9.9%) *p* = 0.04.

### HIV-1 drug mutation patterns

**Figure 3:**
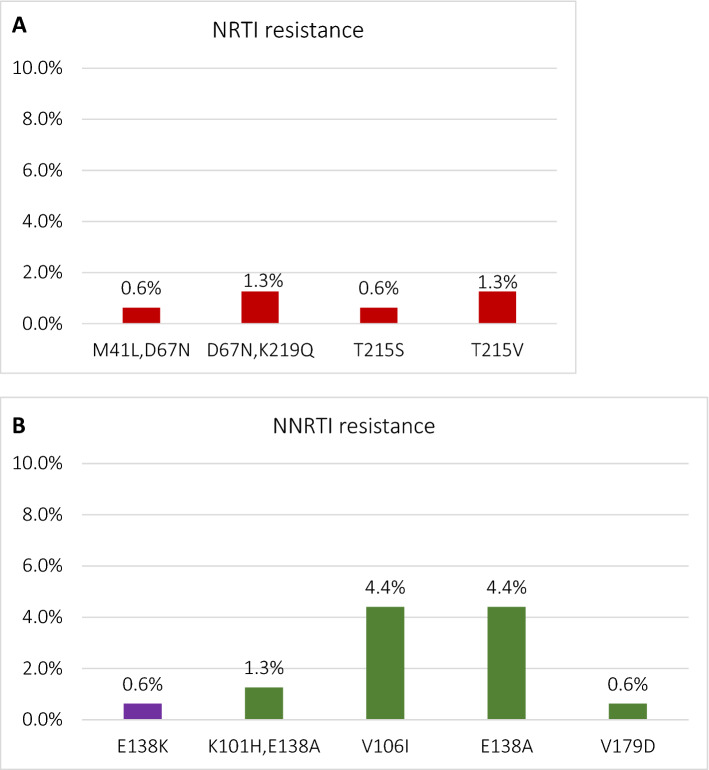

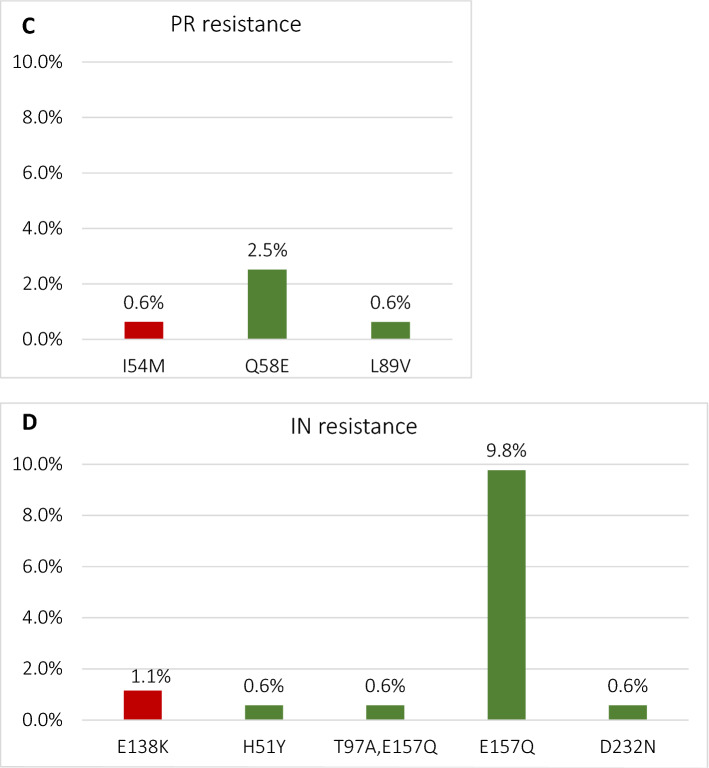
Frequency of major and transmitted drug resistance mutations (red) and variants associated with decreased drug susceptibility (Stanford HIV DB, score ≥ 10, green) for nucl(t)eoside reverse transcriptase (**a**), non-nucleoside reverse transcriptase (**b**), protease (**c**) and integrase (**d**) inhibitors. As non-nucleoside reverse transcriptase E138A mutation is not included in the tDRM list, but is associated with significant reduction of susceptibility to rilpivirine, it was marked in violet.

Based on the Stanford Genotypic Resistance Interpretation Algorithm major or non-accessory NRTI drug resistance was observed in 6 (3.8%) sequences, PI or NNRTI resistance in one (0.6%) case each, and INSTI in two (1.1%) sequences. Additionally, accessory mutations were observed in 5 (3.1%) PI, 17 (10.7%) NNRTI and 20 (11.5%) IN sequences (Fig. 3a–d).

Transmitted drug resistance mutations (tDRM), based on WHO mutation list, were observed in one (0.6%) protease (L54M mutation), 6 (3.8%) NRTI (mutations in the codon positions: M41L, D67N, T215S/V, K219Q) and two (1.1%) (both E138K mutations) integrase sequences (supplemental table 2). No NNRTI tDRM were observed. E157Q polymorphism was the most prevalent (9.8%) resistance associated variant in the analysed dataset and was notably more common among female individuals (n = 4, 50.0%) compared to 8.4% (n = 14) among males, *p* = 0.004. Additionally, we have analyzed the distribution of the L74I polymorphism which was observed in 20 (11.5%) of the integrase sequences, being more prevalent in subtype A samples (n = 16, 94.1%) compared to 2.6% (n = 4) in subtype B (*p* < 0.0001) (supplemental table 3. There were no other significant differences in the drug resistance variant distribution (analyzed for age, gender, donor category, HIV-1 subtype and Fiebig stage or time from the last donation).

### Phylogenetic analyses

**Figure 4:**
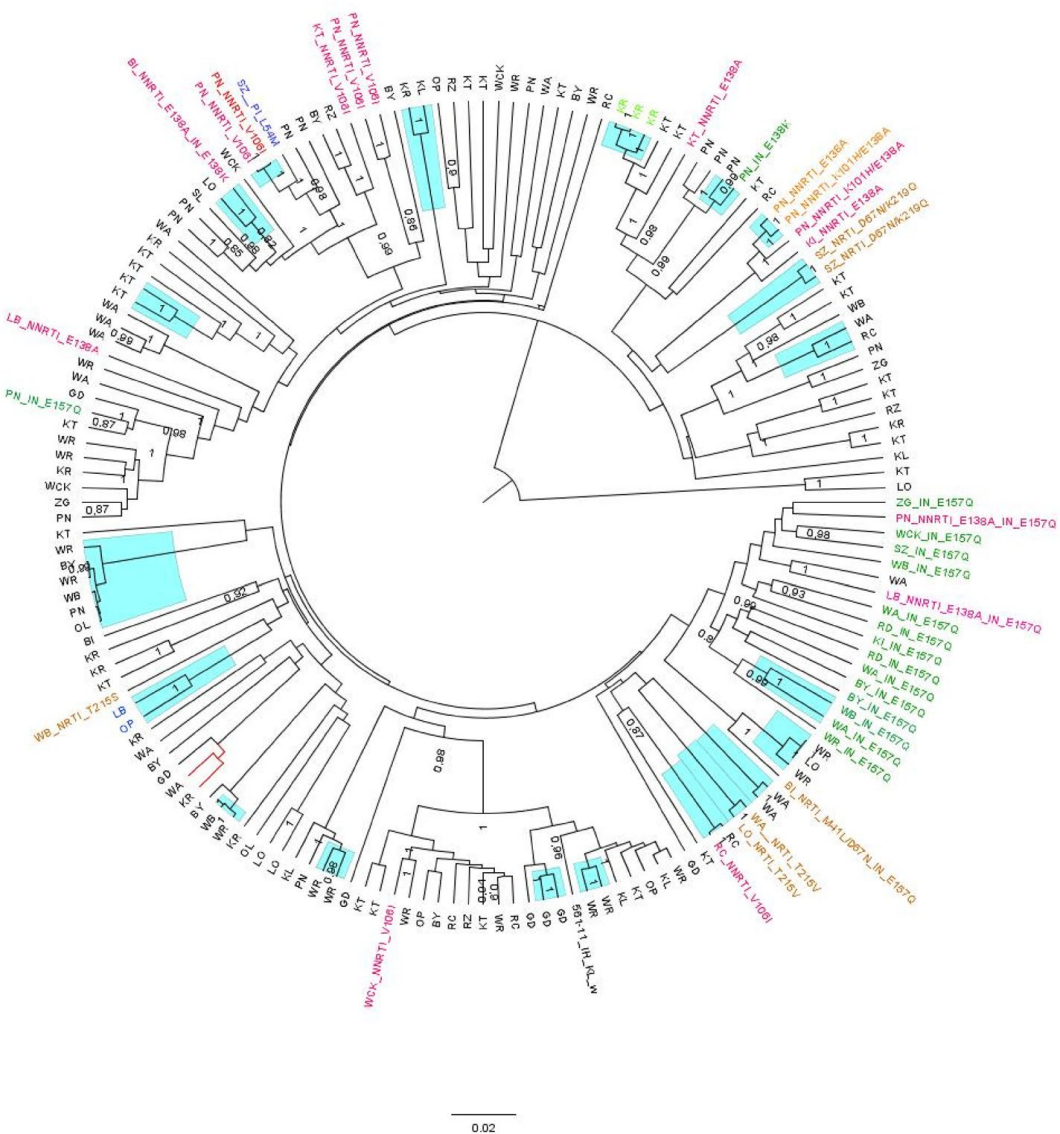
MCMC tree showing the relationship between clusters and mutations and city of diagnosis for subtype B with the inclusion of the protease/reverse transcriptase region of the of the subtype B/A6 recombinant (tree branches marked in red). Transmitted drug resistance substitutions were color-coded and included at the external taxonomical units: brown—resistance against nucleoside reverse transcriptase inhibitors, red—resistance against non-nucleoside reverse transcriptase inhibitors, blue – resistance against protease inhibitors, green—resistance against integrase inhibitors . Clusters have been indicated on the tree with magenta highlight using < 1.5% genetic distance and > 90% branch support.

Clustering was assessed using Bayesian inference, with genetic distance of 1.5%, separately for subtype A and B with 9 (50%) and 44 (26.2% %) of sequences, respectively contained within transmission clusters. There were 16 sequence pairs and 3 (two containing 3 sequences, one with 6 sequences) clusters identified for subtype B and three (all with 3 A6 sequences) for subtype A (Fig. 4, supplemental Fig. 1). It should be noted that the two identified sequences with B/A6 recombinants, proved to be a sequence pair with high similarity both for the protease/reverse transcriptase (red branches in the Fig. 4) and integrase coding regions (red branches in the supplemental Fig. 1). As L74I variant was present in virtually all A6 sequences it was also observed within the identified clusters. In subtype B one cluster with NNRTI K101H/E138A mutation was observed, in four sequence pairs there was also evidence of the shared resistance patterns (NRTI: D67N/K291Q and T215V, NNRTI: V106I, integrase: E157Q).

## Discussion

This study presents the novel data on the HIV-1 subtyping and patterns of drug resistance variants among blood donors from Poland collected in the years 2009–2017. No similar study was performed in the country; moreover the added value of this dataset is the analysis of not only HIV-1 PR/RT but also integrase coding regions. Sampling obtained for sequencing included majority of available samples from the donors with positive HIV molecular test in the country for the above timeframe, therefore may be considered representative for entire population of Polish blood donors.

Subtyping patterns remain in line with the previous data published for the region, with the highest prevalence of the subtype B followed by the subtype A^11,13,14,35^. Of note, in this study we identified 14 sub-subtype A6 sequences and three unique recombinants containing these variants, confirming its import from Russia and Ukraine, most likely by immigration^36^. Interestingly, non-subtype B frequency, especially A6 was associated with repeat donors. This is indicating the circulation of this variant in Polish population adding to the subtype complexity. Additionally, we have identified three recombinants between A6 and B subtype, with a pair of B/A6 sequences showing high similarity despite diagnosis in the distant centers, which may indicate formation of the novel circulating recombinant form. No circulating recombinant forms between A6 and B variants have been described so far. Furthermore, L74I polymorphism was almost invariably (94.1%) present in A6 sequences. This polymorphism, albeit not included in the drug resistance interpretation algorithms, was associated with increased risk of the virologic failure among patients infected with A6/A1 variants treated with long acting cabotegravir/rilpivirine in the ATLAS 2 M study. Further increase in the frequency of A6 sub-subtype in Poland may negatively affect the future virologic response rates to these injectable agents, and underscores the necessity for subtyping and resistance testing prior to introduction of this combination^27^. We have also observed high frequency of transmission clusters calculated with the genetic distance of 1.5%, however DRM were infrequent among closely related sequences. Clustering is a common phenomenon among HIV sequences, also frequently observed among subtype A infected individuals in Europe^1,37,38^.

In general, data on transmitted drug resistance and HIV subtyping patterns are not collected systematically, especially in the region of the central and eastern Europe and as such this dataset provides an important insight on this issue. This is also the first study reporting on the integrase resistance patterns among European blood donors. In the recert reports, frequency of protease/reverse transcriptase DRM among blood donors ranged from 14% in Catalonia^11^, 12.1–13.2% across Chinese provinces^39,40^, 11% in Brazil^41^, however transmission of major DRMs remains infrequent. This is in line with presented data, with frequency of major, non-accessory drug resistance variants for protease, reverse transcriptase or integrase being low, not exceeding 5% for each drug class. As this is a first study on HIV drug resistance among blood donors in the country, no previous patterns in this group may be compared. However, in the largest Polish study published so far on the 833 antiretroviral naïve cases transmitted drug resistance to PR and RT was observed in 9% of sequences, being the most common for NRTI (5.8%), followed by PI 2.0% (2%) and NNRTI (1.2%) mutations, with the highest frequency among heterosexually infected individuals (13.4%) and MSM (8.3%). These frequencies are slightly higher than the frequencies observed in the current study. Moreover, there is an emerging signal for the transmission of the non-polymorphic integrase mutation (E138K), previously not observed in the country^15^. As expected the most common NRTI DRMs were thymidine analog mutations, namely M41L, D67N, T215S/V, K219Q, associated with high levels of resistance to zidovudine, but also affect the abacavir and tenofovir sensitivity. Both these agents remain the cornerstone of the first-line treatments according to the recent national and European guidelines^42,43^. In one sequence the NNRTI non-polymorphic E138K mutation, associated with reduced RPV susceptibility, was found. The remining observed NNRTI DRMs were accessory, potentially reducing susceptibility to etravirine or rilpivirine (E138A). We have previously observed the similar frequency (5.3%) of the rilpivirine associated DRMs with E138A and E138G being the most common DRM^44^. On the other hand, frequency of the V106I variant, which may be affecting doravirine susceptibility was higher (4.4%) than in reference data (0.8%) from Italy and France published by Soulie et al.^45^.

In the integrase region, the most common (9.8%) polymorphism was E157Q, which is usually selected in patients receiving raltegravir or elvitegravir, but not associated with significant effect on the integrase treatment efficacy^46^. This variant may reduce integrase susceptibility if present in combination with other DRMs within this region, especially R263K^47–49^. It was previously observed that in Poland polymorphism was frequent (21%) especially among females, people with history of injection drug use and hepatitis C coinfection^15^. Blood donor regulations exclude patients with history of drug use or HCV coinfection, however in the current study association with female gender was confirmed.

This study also adds valuable information on the recency of HIV infection among blood donors in Poland, reflected by the Fiebig stages at HIV diagnosis. For this purpose, referral to the infection stages labelled as ‘acute’ (Fiebig I), ‘recent’ (Fiebig II–IV) or ‘established’ (Fiebig V–VI) is commonly used^50^. We have noted, that stages associated with recent infection were observed among 9.9% repeat donors, increasing to 40.5% if the stage V was added to the calculations. This is in line with the previous reports from Poland for the years 2001–2007^51^, however in our study calculation of HIV recency was based solely on the HIV-RNA, p24 and Western-blot patterns, with no implementation of the Recent Infection Testing Algorithm (RITA) assays. Donor testing is obviously intended to ensure blood safety, but it should not be overlooked that early HIV diagnosis in the cohort of blood donors in the setting of the low populational testing prevalence^52^ allows for the rapid antiretroviral treatment initiation, reduction of infectivity and risk of onward transmissions^21,50^.

Limitations of the study include lack of more detailed data on the transmission routes or the risk among identified blood donors. Also, calculation on the HIV infection based on Fiebig scale might have underestimated early infection frequency. For this purpose testing with RITA algorithm would add valuable data on the duration of the infection; testing of blood donors with this algorithm should be considered for the future^53^.

To conclude, this study provides a valuable insight on the HIV molecular epidemiology among blood donors in Poland. Transmission of drug resistance in this group was infrequent, however possible emergence of integrase resistance was noted. This emphasises the necessity to continue surveillance on the HIV mutation patterns. Moreover, high frequency of A6 subtype was found indicating migration associated introduction of this subtype to Poland with subsequent local spread and emergence of the new recombinants with the dominant subtype B. This increase in the HIV diversity may potentially affect the antiretroviral susceptibility, even in the context of the novel integrase inhibitors such as cabotegravir.

## Supplementary Information

Supplementary files.

## References

[1] Paraskevis D (2019). HIV-1 molecular transmission clusters in nine European countries and Canada: association with demographic and clinical factors. BMC Med..

[2] Bbosa N, Kaleebu P, Ssemwanga D (2019). HIV subtype diversity worldwide. Curr. Opin. HIV AIDS.

[3] Ahmed N (2019). Development of the R263K Mutation to Dolutegravir in an HIV-1 Subtype D Virus Harboring 3 Class-Drug Resistance. Open Forum Infect Dis..

[4] Verhofstede C (2018). Phylogenetic analysis of the Belgian HIV-1 epidemic reveals that local transmission is almost exclusively driven by men having sex with men despite presence of large African migrant communities. Infect. Genet. Evol..

[5] Rogers, L. *et al.* Structural implications of genotypic variations in HIV-1 integrase from diverse subtypes. *Front Microbiol* **9** (2018). 10.3389/fmicb.2018.01754 PMC6083056 30116231

[6] Patino-Galindo JA (2018). Genome-scale analysis of evolutionary rate and selection in a fast-expanding Spanish cluster of HIV-1 subtype F1. Infect. Genet. Evol..

[7] Vinken L (2019). Earlier initiation of antiretroviral treatment coincides with an initial control of the hiv-1 sub-subtype f1 outbreak among men-having-sex-with-men in flanders belgium. Front. Microbiol..

[8] Zhao J (2020). HIV-1 molecular epidemiology and drug resistance-associated mutations among treatment-naïve blood donors in China. Sci. Rep..

[9] Fiedler SA (2019). Effectiveness of blood donor screening by HIV, HCV, HBV-NAT assays, as well as HBsAg and anti-HBc immunoassays in Germany (2008–2015). Vox Sang.

[10] Laperche S, Tiberghien P, Roche-Longin C, Pillonel J (2017). Fifteen years of nucleic acid testing in france: results and lessons. Transfus. Clin. Biol..

[11] Bes M (2017). Epidemiological trends of HIV-1 infection in blood donors from Catalonia, Spain (2005–2014). Transfusion.

[12] Szmulik K, Niedźwiedzka-Stadnik M, Rosińska M (2019). HIV and AIDS in Poland in 2017. Przegl. Epidemiol..

[13] Parczewski M (2016). Distribution and time trends of HIV-1 variants in Poland: characteristics of non-B clades and recombinant viruses. Infect. Genet. Evol..

[14] Parczewski M (2015). Transmitted HIV drug resistance in antiretroviral-treatment-naive patients from Poland differs by transmission category and subtype. J. Antimicrob. Chemother..

[15] Parczewski M, Leszczyszyn-Pynka M, Urbanska A (2017). Differences in the integrase and reverse transcriptase transmitted resistance patterns in Northern Poland. Infect. Genet. Evol..

[16] Smolen-Dzirba J (2013). Transmission of drug-resistant HIV-1 variants among individuals with recent infection in southern Poland. Curr. HIV Res..

[17] Smoleń-Dzirba J (2019). Transmission patterns of HIV-1 non-R5 strains in Poland. Sci. Rep..

[18] Parczewski M (2017). Expanding HIV-1 subtype B transmission networks among men who have sex with men in Poland. PLoS ONE.

[19] Ewa Sulkowska, D. K.-R., Anna Chrzanowska, Izabela Gordziejewska, Martyna Marcula, Aneta Kopacz, Grzegorz Liszewski, Nico Lelie, Magdalena Łetowska, Piotr Grabarczyk, and Polish Working Group on Transfusion-Transmitted Infections in Blood Transfusion System. HIV infection in Polish blood donors from 2005 to 2018 – trends in epidemiology and residual transfusion transmission risk 53. . *Jahrestagung der Deutschen Gesellschaft für Transfusionsmedizin und Immunhämatologie e. V. (DGTI)* (2020).

[20] Fiebig EW (2003). Dynamics of HIV viremia and antibody seroconversion in plasma donors: implications for diagnosis and staging of primary HIV infection. AIDS.

[21] Stekler JD (2018). No time to delay! Fiebig stages and referral in acute HIV infection: seattle primary infection program experience. AIDS Res Hum Retroviruses.

[22] Van Laethem K (2008). A genotypic assay for the amplification and sequencing of integrase from diverse HIV-1 group M subtypes. J. Virol. Methods.

[23] Woods CK (2012). Automating HIV drug resistance genotyping with RECall, a freely accessible sequence analysis tool. J. Clin. Microbiol..

[24] Pineda-Peña AC (2013). Automated subtyping of HIV-1 genetic sequences for clinical and surveillance purposes: performance evaluation of the new REGA version 3 and seven other tools. Infect. Genet. Evol..

[25] Bennett DE (2009). Drug resistance mutations for surveillance of transmitted HIV-1 drug-resistance: 2009 update. PLoS ONE.

[26] Tzou PL (2020). Integrase strand transfer inhibitor (INSTI)-resistance mutations for the surveillance of transmitted HIV-1 drug resistance. J. Antimicrob. Chemother..

[27] Wensing AM (2019). 2019 update of the drug resistance mutations in HIV-1. Top Antivir. Med..

[28] Madeira F (2019). The EMBL-EBI search and sequence analysis tools APIs in 2019. Nucleic Acids Res..

[29] Yebra G (2016). Using nearly full-genome HIV sequence data improves phylogeny reconstruction in a simulated epidemic. Sci. Rep..

[30] Rhee SY (2006). HIV-1 pol mutation frequency by subtype and treatment experience: extension of the HIVseq program to seven non-B subtypes. AIDS.

[31] Paraskevis D (2004). Phylogenetic reconstruction of a known HIV-1 CRF04_cpx transmission network using maximum likelihood and Bayesian methods. J. Mol. Evol..

[32] Posada D (2008). jModelTest: phylogenetic model averaging. Mol. Biol. Evol..

[33] Drummond, A. J., Suchard, M. A., Xie, D. & Rambaut, A. Bayesian phylogenetics with BEAUti and the BEAST 1.7. *Mol. Biol. Evol.* **29**, 1969–1973, doi:10.1093/molbev/mss075 (2012). 10.1093/molbev/mss075 PMC3408070 22367748

[34] Ragonnet-Cronin M (2013). Automated analysis of phylogenetic clusters. BMC Bioinformatics.

[35] Smolen-Dzirba J (2012). Molecular epidemiology of recent HIV-1 infections in southern Poland. J. Med. Virol..

[36] Schlösser, M. *et al.* HIV-1 Sub-Subtype A6: settings for normalised identification and molecular epidemiology in the southern federal district, Russia. *Viruses* **12**, doi:10.3390/v12040475 (2020).

[37] Hanke K (2019). Reconstruction of the genetic history and the current spread of HIV-1 subtype A in Germany. J. Virol..

[38] Araújo PMM (2019). Characterization of a large cluster of HIV-1 A1 infections detected in Portugal and connected to several Western European countries. Sci Rep.

[39] Liang S (2020). The genotype distribution, infection stage and drug resistance mutation profile of human immunodeficiency virus-1 among the infected blood donors from five Chinese blood centers, 2014–2017. PLoS ONE.

[40] Zeng P (2017). The infection staging and profile of genotypic distribution and drug resistance mutation among the human immunodeficiency virus-1 infected blood donors from five Chinese blood centers, 2012–2014. PLoS ONE.

[41] Esashika Crispim, M. A., da Guarda Reis, M.N., Fraiji, N., Bello, G. & Stefani, M. M. A. Detection of human immunodeficiency virus Type 1 phylogenetic clusters with multidrug resistance mutations among 2011 to 2017 blood donors from the highly endemic Northern Brazilian Amazon. *Transfusion* **59**, 2593-2601, doi:10.1111/trf.15347 (2019 10.1111/trf.15347 31119759

[42] European AIDS Clinical Society. Guidelines v 10.1. https://www.eacsociety.org/guidelines/eacs-guidelines/eacs-guidelines.html (2020).

[43] Polish Scientific AIDS Society (Polskie Towarzystwo Naukowe AIDS). Principles of care for the HIV-infected patients 2019 (in Polish) [Zasady opieki nad osobami zakażonymi HIV 2019]. *PTN AIDS, Warszawa. Eko-Press* (2019).

[44] Parczewski M, Urbanska A, Maciejewska K, Witak-Jedra M, Leszczyszyn-Pynka M (2014). Transmitted drug resistance to rilpivirine among antiretroviral-naive patients living with HIV from northern Poland. J. Int. AIDS Soc..

[45] Soulie C (2020). Prevalence of doravirine-associated resistance mutations in HIV-1-infected antiretroviral-experienced patients from two large databases in France and Italy. J. Antimicrob. Chemother..

[46] Saladini F (2017). The HIV-1 integrase E157Q polymorphism per se does not alter susceptibility to raltegravir and dolutegravir in vitro. AIDS.

[47] Ambrosioni J (2019). E157Q integrase strand-transfer inhibitor substitution in patients with acute/recent HIV infection. AIDS.

[48] Pena, M. J., Chueca, N., D'Avolio, A., Zarzalejos, J. M. & Garcia, F. Virological Failure in HIV to Triple Therapy With Dolutegravir-Based Firstline Treatment: Rare but Possible. *Open Forum Infect Dis* **6**, ofy332, doi:10.1093/ofid/ofy332 (2019). 10.1093/ofid/ofy332 PMC6324549 30631792

[49] Anstett K, Cutillas V, Fusco R, Mesplède T, Wainberg MA (2016). Polymorphic substitution E157Q in HIV-1 integrase increases R263K-mediated dolutegravir resistance and decreases DNA binding activity. J Antimicrob. Chemother..

[50] Crowell TA (2020). Novel criteria for diagnosing acute and early HIV Infection in a multi-national study of early antiretroviral therapy initiation. Clin. Infect. Dis..

[51] Rosińska M (2013). High percentage of recent HIV infection among HIV-positive individuals newly diagnosed at voluntary counseling and testing sites in Poland. AIDS Res. Hum. Retroviruses.

[52] Kowalska JD, Ankiersztejn-Bartczak M, Shepherd L, Mocroft A (2018). Cascade of care and factors associated with virological suppression among HIV-positive persons linked to care in the Test and Keep in Care (TAK) project. Infection.

[53] Zhu Q (2020). Identifying major drivers of incident HIV infection using recent infection testing algorithms (RITAs) to precisely inform targeted prevention. Int. J. Infect. Dis..

