# Supplementary material for: Transmitted HIV drug resistance and subtype patterns among blood donors in Poland
Source: Sci Rep. 2021 Jun 17;11:12734. doi: 10.1038/s41598-021-92210-2 (PMC8211697; doi:10.1038/s41598-021-92210-2)
Supplement: Supplementary file 1 — Supplementary files. [file 41598_2021_92210_MOESM1_ESM.docx]

**Supplementary material**

Title: “Transmitted drug resistance and subtype patterns among blood donors in Poland”.

Authors: **Miłosz Parczewski^1*^**, Ewa Sulkowska^2^, Anna Urbańska**^1^,** Kaja Scheibe^1^, Karol Serwin**^1^**, Piotr Grabarczyk^2^

Affiliations:

1. Department of Infectious, Tropical Diseases and Immune Deficiency, Pomeranian Medical University in Szczecin, Szczecin, Poland

2. Institute of Haematology and Transfusion Medicine in Warsaw, Poland

*Correspondence to:

Milosz Parczewski MD, Ph.D.

Pomeranian Medical University, Department of Infectious, Tropical Diseases and Immune Deficiency, Arkońska 4 71-455 Szczecin, Poland

Tel. 0048918139456, fax 0049918139449

e-mail: mparczewski@yahoo.co.uk

Supplemental figure 1. MCMC tree showing the relationship between clusters and mutations and city of diagnosis for subtype A with the inclusion of the protease/reverse transcriptase region of the of the subtype B/A6 recombinant (tree branches marked in red). Transmitted drug resistance substitutions were included at the external taxonomical unit. Clusters have been indicated on the tree with magenta highlight using <1.5% genetic distance and >90% branch support. To emphasize the close genetic distance for the subtype B/A6 recombinant cluster with the genetic distance <3% and >90% branch support is marked in magenta. Gray highlight indicates A6 variants, the outlier branch is the A1 subtype.


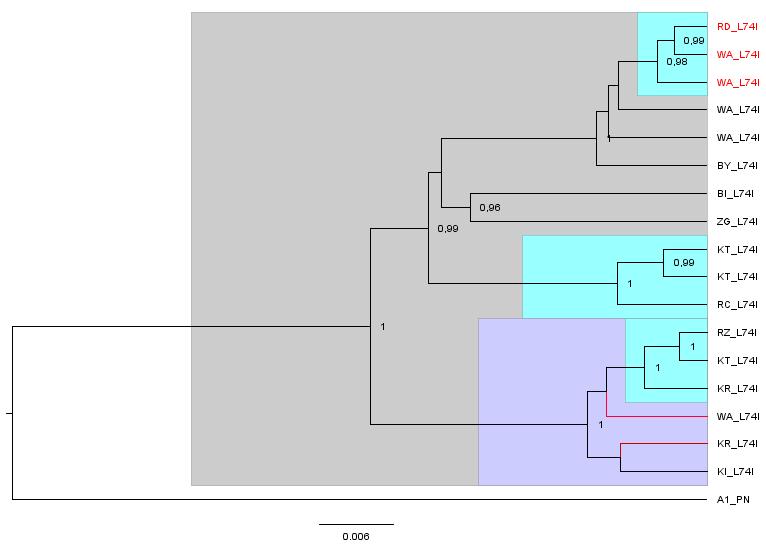


Supplemental table 1. HIV NAAT positive donation rates among Polish donors in the study period

|  |  |  |  |  |  |  |  |  |  |
| --- | --- | --- | --- | --- | --- | --- | --- | --- | --- |
|  |  | **HIV NAAT positive, HIV serology positive** | | | | **HIV NAAT positive, HIV serology negative** | | | |
| **Year** | **Number of donations** | **Number of cases** | **Positive cases per /100.000 donations** | **+95%CI** | **-95%CI** | **Number of cases** | **Positive cases per /100.000 donations** | **+95%CI** | **-95%CI** |
| 2009 | 589398,0 | **54** | 9,161890607 | 6,72 | 11,61 | 1 | 0,495554835 | 0,0299493 | 0,96116 |
| 2010 | 594895,0 | 46 | 7,732456988 | 5,50 | 9,97 | 0 | 0,322879445 | 0 | 0,645759 |
| 2011 | 602861,0 | 51 | 8,459661514 | 6,14 | 10,78 | 4 | 0,982113746 | 0,2580202 | 1,706207 |
| 2012 | 603126,0 | 48 | 7,958536027 | 5,71 | 10,21 | 3 | 0,815879934 | 0,1691614 | 1,462598 |
| 2013 | 601162,0 | 36 | 5,988402461 | 4,03 | 7,94 | 2 | 0,652201439 | 0,0912338 | 1,213169 |
| 2014 | 593102,0 | 38 | 6,406992389 | 4,37 | 8,44 | 1 | 0,49246004 | 0,0297623 | 0,955158 |
| 2015 | 603048,0 | 43 | 7,130444011 | 5,00 | 9,26 | 1 | 0,484337981 | 0,0292714 | 0,939405 |
| 2016 | 629021,0 | 22 | 3,497498494 | 2,04 | 4,96 | 2 | 0,62331588 | 0,087193 | 1,159439 |
| 2017 | 610317,0 | 32 | 5,243176906 | 3,43 | 7,06 | 3 | 0,80626694 | 0,1671683 | 1,445366 |
| total | 5426930 | **370** |  |  |  | **17** |  |  |  |

| Supplemental table 2. Characteristics of individual patients with drug resistance mutations. ^#^ surveillance transmitted drug mutations | | | | | | | | | | | | | |  |  |
| --- | --- | --- | --- | --- | --- | --- | --- | --- | --- | --- | --- | --- | --- | --- | --- |
| Sample ID | Year of donation | Age at donation | Gender | City of diagnosis | Blood donation status | Western Blot pattern | Days since last donation (reccurent donors only) | Fiebig stage | Subtype for integrase region | Subtype for protease/reverse transcriptase region | Major PI DRMs | Accessory PI DRM | NRTI DRMs | NNRTI DRMs | Major IN DRMs |
| 60 | 2011 | 22 | M | Lublin | F | + |  | VI | B | B |  |  |  | E138A |  |
| 252 | 2013 | 25 | M | Warszawa | F | + |  | VI | B |  |  |  |  |  |  |
| 395 | 2011 | 43 | M | Białystok | F | + |  | VI | B | B |  |  | M41L,D67N* |  |  |
| 487 | 2011 | 24 | F | Wrocław | F | + |  | V | B | B |  |  |  |  |  |
| 510 | 2013 | 39 | M | Poznań | R | + | 345 | V |  | B |  |  |  | V106I |  |
| 672 | 2011 | 36 | M | Wrocław | F | + |  | VI | B | B |  |  |  |  |  |
| 795 | 2013 | 22 | M | Wałbrzych | R | + | 163 | VI | B | B |  |  | T215S* |  |  |
| 951 | 2011 | 32 | M | Poznań | R | + | 73 | VI | B | B |  |  |  | V106I |  |
| 971 | 2009 | 29 | F | Poznań | R | + | 89 | V | B | B |  |  |  | E138K |  |
| 1133 | 2009 | 53 | M | Bydgoszcz | F | + |  | VI | B | B |  |  |  |  |  |
| 1324 | 2009 | 24 | M | Poznań | R | + | 1096 | VI | B | B |  |  |  | K101H, E138A |  |
| 1619 | 2012 | 26 | M | Poznań | R | + | 1032 | V | B | B |  |  |  | V106I |  |
| 1663 | 2009 | 39 | M | Bydgoszcz | R | + | 535 | VI | B |  |  |  |  |  |  |
| 1825 | 2014 | 46 | M | Katowice | R | + | 322 | V | B | B |  |  |  | E138A |  |
| 1903 | 2012 | 26 | M | Poznań | R | + | 1424 | VI | B | B |  | Q58E |  |  |  |
| 2095 | 2014 | 34 | F | Radom | R | + | 349 | VI | B | B |  |  |  |  |  |
| 2164 | 2014 | 32 | M | Bydgoszcz | R | + | 449 | VI | C | C |  |  |  | V179D |  |
| 2188 | 2014 | 32 | M | Gdańsk | R | + | 603 | VI | B | B |  | Q58E |  |  |  |
| 2437 | 2014 | 36 | M | Katowice | R | + | 580 | V |  | B |  | Q58E |  |  |  |
| 2445 | 2014 | 32 | M | Poznań | R | - | 121 | II | B | B |  |  |  |  | E138K |
| 2590 | 2015 | 49 | M | Kielce | R | + | 5009 | VI | B | B |  |  |  |  |  |
| 2592 | 2015 | 30 | M | Bydgoszcz | R | - | 68 | I | B | B |  |  |  |  |  |
| 2657 | 2010 | 34 | M | Lublin | F | + |  | VI | B | B |  |  |  | E138A |  |
| 2756 | 2010 | 22 | M | Poznań | R | + | 151 | V | B | B |  |  |  | E138A |  |
| 2794 | 2015 | 27 | M | Poznań | R | + | 136 | VI | B | B |  |  |  |  |  |
| 2884 | 2015 | 41 | M | Wałbrzych | R | + | 226 | VI | B | B |  |  |  |  |  |
| 2919 | 2010 | 27 | M | Warszawa | F | + |  | VI | B | B |  |  |  |  |  |
| 3167 | 2010 | 36 | M | Zielona Góra | F | + |  | VI | B |  |  |  |  |  |  |
| 3213 | 2015 | 31 | M | Szczecin | F | + |  | VI | B | B |  |  | D67N,K219Q* |  |  |
| 3313 | 2015 | 29 | M | Radom | R | + | 528 | VI | B | B |  |  |  |  |  |
| 3475 | 2015 | 21 | M | WCKiK | F | + |  | VI | B | B |  |  |  | V106I |  |
| 3493 | 2015 | 26 | M | Poznań | R | + | 2899 | VI | B | B |  |  |  | V106I |  |
| 3504 | 2015 | 34 | M | Szczecin | R | + | 4305 | V | B | B |  |  |  |  |  |
| 3555 | 2015 | 24 | M | Szczecin | F | + |  | VI | B | B |  |  | D67N,K219Q* |  |  |
| 3709 | 2016 | 27 | M | Poznań | R | + | 170 | V | C | C |  |  |  | E138A |  |
| 3763 | 2016 | 29 | M | Łódź | R | + | 2785 | V | B | B |  |  | T215V* |  |  |
| 3781 | 2011 | 34 | M | Racibórz | R | + | 94 | VI | B | B |  |  |  | V106I |  |
| 3888 | 2016 | 41 | M | Warszawa | F | + |  | VI | B | B |  |  |  |  |  |
| 3938 | 2011 | 27 | M | Poznań | R | + | 584 | VI | B | B |  |  |  | K101H, E138A |  |
| 3963 | 2016 | 37 | M | Zielona Góra | R | + | 147 | VI | B | B |  | Q58E |  |  |  |
| 4223 | 2016 | 62 | M | Wałbrzych | R | - | 61 | I | B | B |  |  |  |  |  |
| 4716 | 2017 | 28 | M | Warszawa | R | + | 797 | VI | B | B |  |  | T215V* |  |  |
| 4866 | 2017 | 33 | F | WCKiK | F | + |  | VI | B | B |  |  |  |  |  |
| 5218 | 2017 | 40 | M | Katowice | R | + | 6633 | VI | B | B |  |  |  | V106I |  |
| 5330 | 2017 | 28 | M | Szczecin | R | + | 187 | VI | B | B | I54M* |  |  |  |  |
| 5514 | 2017 | 27 | M | Białystok | R | + | 110 | VI | B | B |  |  |  | E138A | E138K* |
| 29n | 2006 | 31 | M | Kielce | R | p24 band only | 139 | I | B | B |  |  |  | E138A |  |
| 3017# | 2015 | 26 | M | Katowice | R | + | 74 | V | A6 | A6 |  | L89V |  |  |  |
| #sequence also included in the supplemental table 1. Codes: gender: M- male, F- female, type of blood donation: F – first time donor, R – recurrent donor | | | | | | | | | | | | | | | |

| Supplemental table 3. Characteristics of individual patients with L74I polymorphism in the integrase coding regions | | | | | | | | | | | | |
| --- | --- | --- | --- | --- | --- | --- | --- | --- | --- | --- | --- | --- |
| Sample ID | Year of donation | Age at donation | Gender | City of diagnosis | Blood donation status | Western Blot pattern | Days since last donation (reccurent donors only) | Fiebig stage | Subtype for integrase region | Subtype for protease/reverse transcriptase region | L74I |  |
| 5474 | 2017 | 29 | M | Warszawa | R | + | 192 | V | B | A6 | L74I |  |
| 2495 | 2014 | 43 | M | Kraków | R | + | 228 | VI | B | A6 | L74I |  |
| 2575 | 2010 | 28 | M | Katowice | F | + |  | VI | B |  | L74I |  |
| 264 | 2011 | 35 | M | Warszawa | R | Gp 41 only | 109 | IV | A6 | A6 | L74I |  |
| 1486 | 2014 | 38 | M | Katowice | R | + | 161 | V | A6 | A6 | L74I |  |
| 1581 | 2014 | 32 | M | Katowice | R | + | 130 | V | A6 | A6 | L74I |  |
| 2757 | 2015 | 34 | M | Zielona Góra | R | + | 165 | VI | A6 | A6 | L74I |  |
| 2849 | 2015 | 25 | M | Bydgoszcz | R | Gp41 only | 114 | IV | A6 | A6 | L74I |  |
| 3054 | 2015 | 24 | M | Kielce | R | + | 837 | VI | A6 | A6 | L74I |  |
| 3193 | 2015 | 28 | M | Warszawa | R | + | 498 | VI | A6 | A6 | L74I |  |
| 3319 | 2015 | 29 | M | Radom | R | + | 679 | VI | A6 | A6 | L74I |  |
| 3600 | 2011 | 22 | M | Warszawa | R | + | 42 | V | A6 | A6 | L74I |  |
| 3797 | 2016 | 31 | M | Rzeszów | R | + | 2881 | VI | A6 | A6 | L74I |  |
| 4698 | 2017 | 25 | M | Kraków | R | + | 526 | VI | A6 | A6 | L74I |  |
| 5086 | 2017 | 33 | K | Białystok | R | + | 183 | VI | A6 | A6 | L74I |  |
| 777 | 2013 | 37 | M | Katowice | R | + | 186 | V | B | B | L74I |  |
| 4115 | 2016 | 39 | M | Warszawa | R | - | 304 | I | B | B | L74I |  |
| 1800 | 2012 | 22 | M | Racibórz | F | + |  | VI | A6 | A6 | L74I |  |
| 3267 | 2015 | 25 | M | Military blood service | F | + |  | VI | B |  | L74I |  |
